# The Recombinant Viral Capsid Protein rVP1 Induces Protective Immunity Against Coxsackievirus B3 (CVB3) Lethal Challenges in Balb/c Mouse Model

**DOI:** 10.3390/vaccines14030244

**Published:** 2026-03-06

**Authors:** Manel Ben M’hadheb, Ikbel Hadj Hassine, Mohammed A. Almalki, Mouna Hassine, Jawhar Gharbi

**Affiliations:** 1 Department of Biological Sciences, College of Science, King Faisal University, P.O. Box 380, Al-Ahsa 31982, Saudi Arabia; malmalki@kfu.edu.sa; 2 Department of Virology, Health Sciences College, Western University, 1393 Western Road, London, ON N6G 3K7, Canada; ihadjhas@uwo.ca; 3 Virology and Antiviral Strategies Research Unit UR17ES30, College of Biotechnology, Monastir University, BP 74, Tahar Hadded Street, Monastir 5000, Tunisia; mouhassi@yahoo.fr

**Keywords:** CVB3, recombinant viral protein rVP1, immunogenicity, vaccine, Balb/c mice

## Abstract

**Background/Objectives:** Epidemiological studies have proven that coxsackievirus B3 (CVB3) is the major virus that causes acute and chronic myocarditis and pancreatitis. Currently, there are no antiviral therapeutic drugs or vaccines that are available for use as clinical therapeutics or vaccines. Subunit polypeptides-based vaccines, especially when combined with adjuvants, represent safe and effective vaccine platforms because they are considered to be better immunogens. The viral capsid protein VP1 of CVB3 is the most immunogenic viral polypeptide, providing opportunities for its use in designing subunit polypeptide vaccines. In the present study, we designed and produced a CVB3 vaccine candidate based on the recombinant expression of the major immunogenic viral protein VP1 of a wild-type CVB3 strain. **Methods:** We assessed its induced humoral and cellular immune responses and then evaluated its protective immunity against pathogenic CVB3 strain challenges in a Balb/c mouse model. Neutralizing specific antibodies and cytokine interferon gamma (INF-γ) production were determined in the sera of both prime- and prime-boost-immunized mice with the vaccine candidate. **Results:** Our results demonstrate that the recombinant rVP1 expressed in a eukaryotic insect cell baculovirus vector system elicited cellular and humoral immune responses, protecting Balb/c mice from lethal challenges. **Conclusions:** Hence, the vaccine produced based on the recombinant expression of VP1 is a promising and potential candidate against natural CVB3 infections.

## 1. Introduction

Coxsackievirus B3 (CVB3), belonging to the family *Picornaviridae*, is a human enterovirus that contains a small RNA molecule as its genome. Human enteroviruses are organized into four different species. *Enterovirus A* to *D* are ubiquitous infectious agents and infect many organs within human body systems. Coxsackieviruses (CVBs) are mainly transmitted fecal-orally [1]. The association of CVBs with pancreas and heart diseases is evident. Several studies have demonstrated that CVBs are the most common cause of myocarditis, pancreatitis, Type 1 diabetes (T1D) and chronic cardiomyopathy [2,3]. The genome of CVB3 is a positive-stranded RNA segment that is approximately 7500 nucleotides long. The unique ORF (Open Reading Frame) is flanked by two non-translated regions (NTRs) in 5′ and 3′ and terminates with a poly (A) tract. The two regions in the 5′NTR and 3′NTR are highly conserved in RNA sequence and structure. These regions have been revealed to be important for virus replication, translation, and pathogenesis. The viral genome of CVB3 encodes a single ORF (Open Reading Frame) to finally synthetize a viral polyprotein during translation that is composed of 2185 amino acids. This polyprotein is cleaved by viral enzymes into viral sub-proteins P1, P2, and P3. These sub-proteins are then cleaved by viral proteases, producing several structural viral proteins (VP1, VP2, VP3, and VP4) and viral enzymes. The structural capsid proteins, VP0, VP1, and VP3, form protomers, and five protomers form a pentamer. The icosahedral viral capsid with a diameter of ~30 nm is finally composed of 12 pentamers. Whereas VP4 is an internal protein, the other viral proteins, VP1 to VP3, are exposed on the viral particle surface [4,5]. Several studies have revealed that the VP1 viral protein is the most exposed on the viral capsid and contains the most neutralizing epitopes and virulence determinants, including major ones [6,7]. This viral protein has been widely used to investigate virus evolution, diagnosis, and pathogenesis [8,9]. By using intraperitoneal or oral routes of infection, several studies have demonstrated that wild-type CVB3 and pathogenic strains replicate in mouse models and induce murine diseases, which mimic those in humans [10,11]. The experimental intraperitoneal Balb/c mouse model was useful for CVB3 pathogenesis research, although the natural route of CVB3 transmission is oral.

The expression of viral proteins can be realized in several living or cell-free expression systems, after which a viral capsid can be reconstructed. The systems used for the expression and the production of recombinant proteins have been developed using the prokaryotic and eukaryotic cells, allowing the production of target viral proteins both at laboratory and industrial scales [12]. Recombinant proteins are gaining in popularity in the field of preventive medicine and to date, a wide range of subunit-based candidate vaccines have been developed for immunization against various infectious agents. Subunit polypeptide-based vaccines are highly immunogenic and elicit both the humoral and the cellular immune responses by pathways different from those elicited by conventional inactivated and live-attenuated vaccines [13,14,15,16]. The potential technological advantages of recombinant vaccines have led researcher teams to choose suitable and potential antigen proteins, have demonstrated exceptional safety and efficacy, making subunit-based vaccine technology an appealing approach for the prevention of viral infections. Traditional vaccine technologies have limitations, especially in terms of production efficacy and safety [17,18]. However, recombinant viral proteins technology can be applied more broadly than traditional technologies including viruses that do not grow in cell cultures. Recently, recombinant proteins have attracted considerable attention for their ease of synthesis, excellent biocompatibility, and tunable structural design. These polypeptides have found wide application in biomedical and material sciences and are increasingly recognized as promising platform for designing of vaccines [19,20].

Although vaccination against coxsackieviruses, and especially the CVB3 serotype, could reduce the incidence and the severity of acute and chronic human cardiac and pancreas diseases caused by this virus, there are currently no clinically therapeutic or prophylactic drugs or vaccines. In the current study, we report our contribution towards the design and the development of a recombinant viral protein rVP1 based vaccine. Our results highlighted the protective cellular and humoral immune responses induced by the intraperitoneal administration of the vaccine candidate in Balb/c mice model.

## 2. Materials and Methods

### 2.1. Mice Experiment Ethics

The mice model study of the present study has been carried out in accordance with the recommendations of the King Faisal University Committee of Ethics (Deanship of Scientific Research KFU-DSR) as described in the DSR-Ethics guide for animal experimentations (Agreement# KFU-REC-OCT-ETHICS247). The Ethics Committee supervised the protocol. All mice experiments were conducted making efforts to minimize suffering Balb/c mice during anesthesia and blood collection. Balb/c mice were bred in the college of science animal facility. All described mice experiments complied with the recommendations of the Committee of Ethics of the DSR. The Ethics Committee approved and supervised the designed protocol.

### 2.2. Cells, Media and CVB3 Virus Strains

HEK293T cells (ATCC CRL-11268) were cultured in MEM (Invitrogen, Carlsbad, CA, USA) supplemented with 7% of FCS (Invitrogen, Carlsbad, CA, USA), 1% L-glutamine (Invitrogen, Carlsbad, CA, USA), 50 µg/mL of streptomycin (Invitrogen, Carlsbad, CA, USA), 25 UI/mL of penicillin (Invitrogen, Carlsbad, CA, USA), 1% non-essential amino acids (Invitrogen, Carlsbad, CA, USA) and 0.1% of Amphotericin B (Invitrogen, Carlsbad, CA, USA) in a humidified incubator with 5% CO_2_ at 37 °C. Viral supernatants were collected, clarified by centrifugation and stored at −80 °C until using. For recombinant rVP1 production in eucaryotic system, Sf9 insect cells (*Spodoptera frugiperda*, ATCC CRL-1711) were cultured in suspension and grown at a non-humidified shaker at 28 °C in TC-100 Insect Medium (Invitrogen, Carlsbad, CA, USA) supplemented with 10% FCS (Invitrogen, Carlsbad, CA, USA), 10,000 UI/mL streptomycin (Invitrogen, Carlsbad, CA, USA), 50 mg/mL penicillin (Invitrogen, Carlsbad, CA, USA), 25 UI/mL Amphotericin (Invitrogen, Carlsbad, CA, USA). The cardiopathogenic CVB3/Nancy wild type and the myocarditic CVB3/20 strains were kindly provided by Professor Steven Tracy from the Enterovirology laboratory, College of Medicine, University of Nebraska Medical Center (UNMC, Omaha, NE, USA) were propaged and maintained on HEK293T cell cultures. Due to their reliable ability to cause heart and pancreas inflammations and subsequent myocarditis and pancreatitis in susceptible mice, the CVB3/Nancy and CVB3/20 strains served as a crucial laboratory model for studying the pathogenesis of viral heart disease and evaluating potential therapies or vaccines. The CVB3/20 strain was first isolated from selenium-deficient donor mice. Virus titers were determined using the limiting dilution assays for 50% TCID according to the Reed and Muench assay [21].

### 2.3. Production, Purification and Characterization of the rVP1 of CVB3

The baculoviral transfer vector pFastBac1 (Invitrogen, Carlsbad, CA, USA) containing the total gene encoding the CVB3 Nancy wild type VP1 capsid protein, previously cloned was used in this study. Generation of recombinant viral protein rVP1 was conducted using the recombinant baculovirus cultured in Sf9 infected insect cells (Invitrogen, Carlsbad, CA, USA) and the Bac-to-Bac vector system. Briefly, Sf9 cells cultured in media without FCS at 30 °C were co-infected with recombinant baculoviruses containing the total gene of the CVB3 VP1 capsid protein at a multiplicity of infection (MOI) of 1 TCID_50_/cell. Sf9 cells were lysed using a lysis buffer (50 mM Tris and 2 mM MgCl_2_ [pH 7.5]) at a cell density of 2 × 10^7^ cells/mL. Sf9 cells were then lysed by conducting three freeze–thaw cycles in nitrogen liquid. Cell lysates were then mixed with 50 U/mL benzonase (Invitrogen, Carlsbad, CA, USA) with the addition of 150 mM NaCl for 1 h at 37 °C. Cell supernatants expressing rVP1 protein were collected, clarified via Sartoclear dynamics Lab filtration (Sartorius, Gottingen, Germany) and purified to eliminate cellular and baculoviral residual contaminants successively by centrifugation at 5000× *g* for 20 min and by ultracentrifugation at 200,000× *g* for 60 min using discontinuous sucrose gradients with SW55 Ti Swinging-Bucket Rotor (Backman Coulter, Brea, CA, USA). Fraction located at 35% sucrose interface, representing generated rVP1 product was subjected for further purification with ion-exchange chromatography. Purification of ultra centrifugated products involved ion-exchange chromatography using HiLoad™ 26/10 Q Sepharose High Performance and HiTrap Q Sepharose XL columns (Cytiva, MA, USA) to remove impurities. The final concentrated rVP1 preparations were stored in Tris buffered saline (40 mM Tris–HCl, pH 7.4, 10 mM MgCl_2_, 0.2 M NaCl + 0.1% Tween 80) at −80 °C. Identification and purity of the ultracentrifuged and purified CVB3 viral protein rVP1 were analyzed using multiple biochemical and biophysical assays. rVP1 product was first characterized by running on 12% SDS-PAGE gels (Bio-Rad, CA, USA) and then analyzed by WB (Western blotting, Bio-Rad, CA, USA) using the mouse monoclonal antibody (mAb) anti-enterovirus VP1 (clone 5-D8/1, Dako, Glostrup, Denmark) at dilution of 1:4000, followed by incubation with a secondary goat antibody coupled to AlexaFluor 488 against mouse immunoglobulins (Dako, Glostrup, Denmark) diluted at 1:15,000. Stained Sf9 infected cells were daily examined using immunofluorescence microscope (Zeiss Axio, Oberkochen, Germany, Imager 2). Purity of the rVP1 product was evaluated in triplicate assays by residual dsDNA content quantification using the Quant-iT dsDNA high-sensitivity kit (Invitrogen, Carlsbad, CA, USA) as recommended by the company. This DNA assay kit is highly selective for double-stranded DNA, and in the ranges of 0.1–100 ng DNA. In addition, the product purity was evaluated by the detection of residual baculovirus particles using mouse monoclonal anti-goat anti-gp64 mAb (Dako, Glostrup, Denmark) diluted at 1:2000, followed by incubation with the secondary goat anti-mouse antibody (Dako, Glostrup, Denmark) diluted at 1:40,000.

### 2.4. Balb/c Mice Immunization and Challenge Strategies

Four-week-old female Balb/c mice were treated according to the general ethic rules of KFU-DSR ethical committee and maintained under specific pathogen-free conditions with unlimited food and water access. Balb/c mice were monitored daily for clinical signs, body weight, and mortality. Mice were divided randomly into 8 groups (G1 to G8). Each group with 6 mice (n = 6):

Group 1 and Group 2 mice received a prime intraperitoneal inoculation with 12 µg of rVP1 based-vaccine at day 0, then challenged successively at week 4 with 3.2 × 10^5^ TCID_50_ of CVB3 wild type and 1.28 × 10^5^ TCID_50_ of CVB3/20 strains.

Group 3 and Group 4 mice received first a prime-boost regimen at day 0 and week 3 with 12 µg of rVP1, then challenged at week 4 successively with the same doses of CVB3 wild type and CVB3/20 strains.

Group 5 mice received just a prime-inoculation at day 0 with 12 µg of rVP1 based-vaccine candidate.

Group 6 and Group 7 mice were used in this study as positive control. Mice were inoculated with PBS at days 0 and week 3, then challenged at week 4, respectively, with CVB3 wild type and CVB3/20 strains.

Group 8 Balb/c mice, representing the naive control group received only a prime-inoculation at day 0 with PBS.

Viral doses used during the lethal challenges were chosen according to a similar previous study in the same mice model [7]. All mice groups were monitored daily during the study period (from day 0 to week 6) for body weight changes and mortality. We used in our study 6 mice (n = 6) in each animal group. The death of a single mouse corresponds to 83% as percentage of mice survival. Heart and pancreas organs were collected from euthanized mice at the end of the study (Week 6). Organ tissues were rinsed and stored at −80 °C in nitrogen liquid for further investigations. At weeks 1, 2, 3, 4, and 5 post prime-inoculation, blood samples were collected from mice tails for interferon gamma (INF-γ) quantification and neutralizing antibody titrations assays. At week 6 (End of the study), blood samples were collected directly from hearts during the final mice euthanasia.

### 2.5. Neutralizing Antibody Titration Assay

HEK293T cells were cultured in 96-well microtiter plates at 6 × 10^3^ cells/well in 50 µL of medium (MEM with 7% of FCS, 1% L-glutamine, 50 µg/mL of streptomycin, 25 UI/mL of penicillin, 1% non-essential amino acids and 0.1% of Amphotericin B). HEK293T cells were then incubated for 16 to 20 h at 37 °C at 5% CO_2_. Serums of immunized mice groups were inactivated at 56 °C for 30 min, then diluted at serial two-fold dilutions with bovine serum albumin (BSA). Titrations of specific neutralizing antibodies were calculated using the micro-neutralization assay. The method is based on HEK293T cell viability as described previously [22]. Briefly, 50 µL of two-fold serial serum dilutions were incubated, respectively, with 50 μL of 100 TCID_50_ CVB3 wild type or CVB3/20 strains at 37 °C for 1 h, followed by the addition of HEK293T cells (concentration of 6 × 10^3^ cells/well). After 46 h of incubation at 37 °C, HEK293T cell viability was evaluated with alamar-blue reagent. Neutralizing antibodies were evaluated by calculating the inhibition concentration titers (IC_50_) from sample-specific neutralization curves. The highest dilution of serum that inhibited the viral cytopathic effect corresponds to the neutralizing antibody titer.

### 2.6. IFN-γ Concentration Assay

Sera were collected at different weeks 1 to 6 post-prime immunization from the immunized and challenged Balb/c mice groups and from the naive Balb/c mice group. The IFN-γ concentrations were calculated by ELISA assay, using a murine IFN-γ platinum ELISA kit (Invitrogen, Carlsbad, CA, USA) as described previously [22].

### 2.7. Viral Titration and Tissues Histopathology Assays

Heart and pancreas tissues from euthanized mice at week 6 were removed and rinsed with PBS. For viral titration by TCID assay, Snap-frozen tissue portions were weighted, crushed using a tissue homogenizer (Qiagen, Germantown, MD, USA), clarified at 2000× *g* for 10 min at 4 °C, suspended in PBS with 1% antibiotics and finally stored at −80 °C for viral titrations. HEK293T cells were cultured on a six-well plate at 3 × 10^6^ in cell culture medium (MEM, Invitrogen, Carlsbad, CA, USA) with 7% of FCS, 1% L-glutamine, 50 µg/mL of streptomycin, 25 UI/mL of penicillin, 1% non-essential amino acids and 0.1% of Amphotericin B in 5% CO_2_ incubator at 37 °C for 24 to 48 h. Culture supernatants from clarified snap-frozen mice tissues were inoculated to HEK293T cells. Cell cultures were examined daily using light microscopes for the development of cytopathic effect. Virus titers were evaluated by the Reed and Muench assay [21] and expressed as TCID_50_ by mg tissue values. For organ histopathology assay, Balb/c mice were euthanized at the end of the study (weeks 6 post-prime inoculation). Heart and pancreas tissues were fixed in formalin for paraffin embedding. Tissue serial of 3 to 5 μm thick sections were then stained using eosin and hematoxylin (HE) and finally examined by light microscope for the presence of lymphocyte infiltrations and inflammatory lesions.

### 2.8. Statistical Analysis

We used Student’s *t* test to establish statistical significance of three (n = 3) replicated experiments (*p* < 0.05) using Graphpad Prism 6 Version 6.02.

## 3. Results

### 3.1. Biochemical and Biophysical Characterization of the Recombinant rVP1 Protein

**Figure 1:**
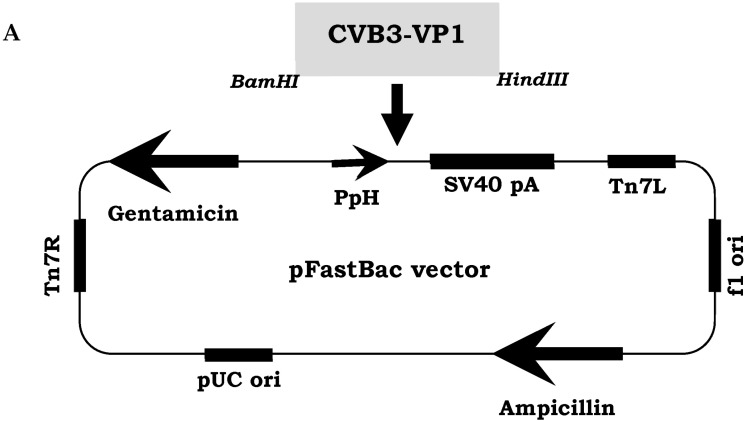

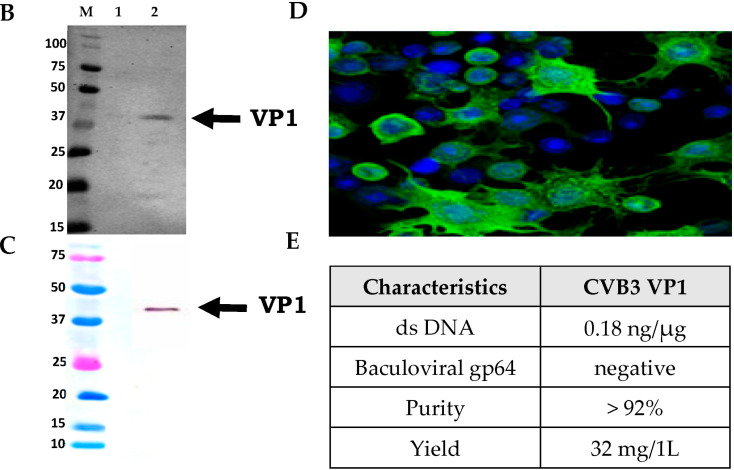
Identification and characterization of the CVB3 rVP1. (**A**) The schematic representation of the VP1 viral protein of CVB3 wild type strain cloned in the Bac-to-Bac vector system as described in Section 2. (**B**) SDS-PAGE and (**C**) WB analyses of the purified recombinant rVP1 are shown. M, Molecular Weight marker; 1, the negative control and 2, the revealed recombinant viral protein rVP1 of CVB3 wild type visualized with arrows. WB analysis revealed that the produced rVP1 was recognized by 5D-8/1 mAb. (**D**) IF analysis of insect Sf9 infected cells. IF assay revealed the presence of CVB3 rVP1 in the Sf9 cells cytoplasm. Infected cells were stained by the 5-D8/1 specific mAb and the conjugated goat anti-mouse coupled to AlexaFluor 488. The nuclei of cultured cells are stained with DAPI coloration (Blue), Magnification 100×. (**E**) Characteristics of the purified rVP1, including the rVP1 purity, dsDNA and baculovirus gp64 glycoprotein contents and the total yield of purified rVP1 product.

The recombinant viral protein rVP1 of CVB3 wild type strain, was produced and purified using the Bac-to-Bac recombinant baculovirus system as described in Section 2. The baculoviral transfer vector pFastBac1 (Invitrogen, Carlsbad, CA, USA) contained expression cassette of the CVB3 VP1 protein (under the strong polyhedron promoter PpH) (Figure 1A). Recombinant baculoviruses were cultured in Sf9 (*Spodoptera frugiperda*) insect cells. CVB3 rVP1 was expressed in Sf9 cells at a MOI (Multiplicity of infection) of 1 TCID_50_/cell. Three days post infection, culture supernatants containing rVP1 were ultracentrifuged, collected, and purified as described in Section 2. Purified rVP1 was identified by SDS-PAGE, Western blot (WB) and Immunofluorescence (IF) staining. Results of the SDS-PAGE assay revealed that the molecular weight (MW) of the produced recombinant viral protein rVP1 matched with the theoretical prediction, which is equal to 33–34 kD (Figure 1B). Using the WB method, rVP1 was recognized by the monoclonal antibody (mAb) 5D-8/1 directed against enterovirus VP1 viral protein, matching with the same MW (Figure 1B).

For further additional quality assessments of produced rVP1, we evaluated the purity of product stocks by residual dsDNA content quantification and by the detection of residual baculovirus particles. Results demonstrated the absence of residual dsDNA contaminants deriving from the cell culture process. The residual dsDNA level was evaluated at 0.18 ng/μg, that is considered acceptable (acceptation limit is 10 μg/dose) according to a previous study [23]. In addition, our results of the detection of residual baculoviral particles using WB assay and anti-gp64 mAb were negative. The yield of the rVP1 was evaluated to be 32 mg/1 L and the purity was more than 92% (Figure 1D). Our results confirmed that the produced rVP1 stock is pure, with a sufficient quantity, free of baculoviral gp64 glycoprotein and with residual dsDNA level that is considered suitable to be useful for further immunization experiments in Balb/c mice model.

### 3.2. Neutralizing Antibodies and IFN-γ Cytokine Responses Induced by the CVB3 rVP1 Immunization in Balb/c Mouse Model

**Figure 2:**
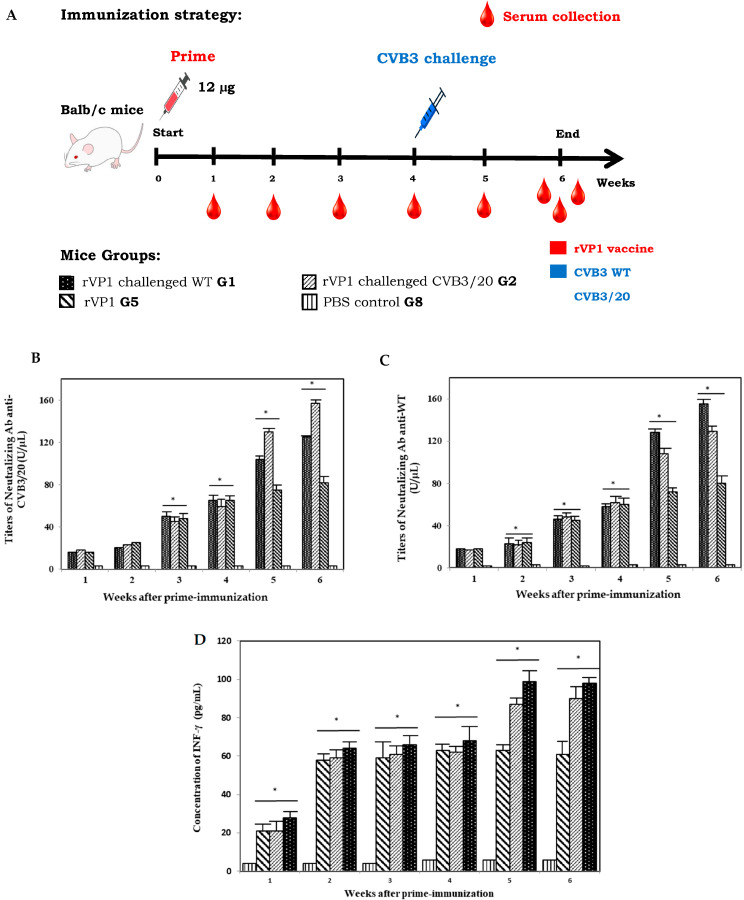
Humoral (Specific neutralizing Ab) and cellular (IFN-γ cytokine) immune responses induced by the rVP1 vaccine candidate in Balb/c mouse model. (**A**) Immunization schedule, Balb/c mice (n = 6, per group) were prime immunized at day 0 with 12 µg of purified rVP1 vaccine candidate, then challenged at week 4 with 3.2 × 10^5^ TCID_50_ of CVB3 WT strain (G1) or with 1.28 × 10^5^ TCID_50_ of CVB3/20 strain (G2). Mice Group 5 (G5) received only a primary inoculation with CVB3 rVP1 at day 0 without any challenge. (**B**) Titers of specific neutralizing Ab anti-CVB3 WT and (**C**) anti-CVB3/20 produced in the sera of mice groups (G1, G2, G5 and G8) were measured at weeks 1 to 6 post prime-immunization. (**D**) Concentration of IFN-γ cytokine in the sera of mice groups (G1, G2, G5 and G8) at weeks 1 to 6 post prime-immunization. The concentrations of IFN-γ cytokine were measured by ELISA assay as described in Section 2. The data shown are the meaning of +/− SD from three replicated experiments (n = 3). * *p* < 0.05 compared vs. control, Student’s *t* test.

To investigate whether the produced CVB3 rVP1 vaccine candidate can induce a humoral and cellular response in vivo, we evaluated first the titers of specific neutralizing antibodies and the cytokine IFN-γ quantifications in sera collected from mice groups 1, 2, 5 and 8 (G1, G2, G5 and G8) from week 1 to week 6 (Figure 2A). Mice groups G1 and G2 were immunized intraperitoneally at day 0 with 12 µg of purified vaccine candidate and then challenged successively at week 4 with CVB3 wild type (WT) and CVB3/20 strains. Mice group G5 were not challenged with virus strains as mentioned in Section 2. Mice group G8 control naive mice were, however, injected only with PBS. Specific neutralizing Ab anti-WT and anti-CVB3/20 titer changes and concentrations of IFN-γ were examined and calculated in mice group sera at weeks 1 to 6.

As shown in Figure 2B,C, our results showed that the neutralizing Ab targeting the rVP1 protein titers of all mice groups except the naive control group 8 (G8) demonstrated a significant increase after prime vaccination with rVP1 for both assays (Neutralizing Ab anti-WT and anti-CVB3/20). Interestingly, the level of produced neutralizing Ab (expressed in U/µL) in sera of mice groups challenged at week 4 by CVB3 WT and CVB3/20 (G2 and G3) exhibited a remarkable increase, revealed at weeks 5 and 6 post prime-immunization due to the challenge with WT and CVB3/20 strains.

Results of IFN-γ production in mice sera presented in Figure 2D showed clearly that the IFN-γ concentrations (expressed in pg/mL) among the different immunized mice groups (G1, G2, and G5) increased significantly in comparison with the non-immunized mice group (G8). In the same way, IFN-γ levels revealed an important increase in concentration between 68% and 72% in weeks 5 and 6 post prime-immunization for mice groups challenged at week 4 by CVB3 WT and CVB3/20 (G2 and G3). The evaluation of neutralizing Ab and IFN-γ concentrations of the different mice groups highlighted that immunization with a single dose with the rVP1 vaccine candidate could induce an effective cellular and humoral immune responses in mouse model.

### 3.3. Balb/c Mice Protection Against CVB3 Lethal Challenges by the rVP1 Vaccination

**Figure 3:**
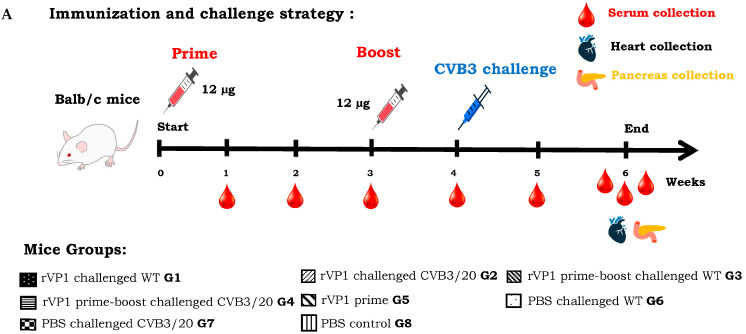

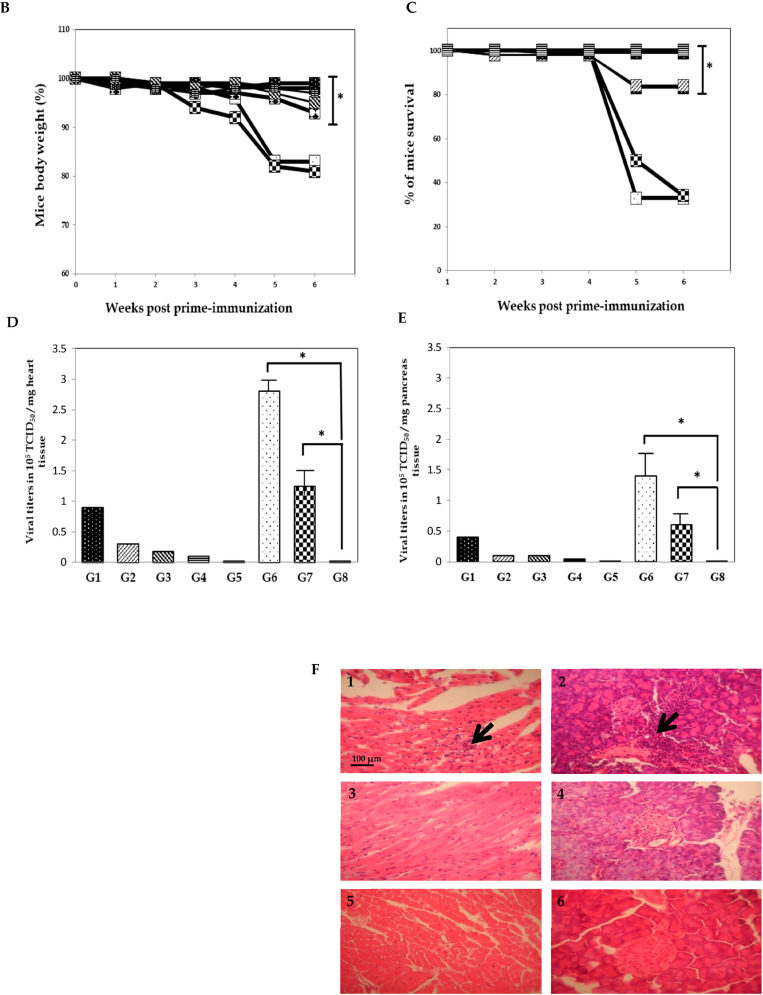
Mice protection by CVB3 rVP1 vaccination against lethal challenges. (**A**) Mice immunization and challenge strategy used in the present study, female Balb/c mice groups (G1–G5) received a prime-immunization at day 0 with 12 µg of purified CVB3 rVP1 vaccine candidate. Mice groups (G1 and G2) were then challenged at week 4 with 3.2 × 10^5^ TCID_50_ of CVB3 WT strain (G1) or with 1.28 × 10^5^ TCID_50_ of CVB3/20 strain (G2). Mice groups (G3 and G4) were boosted at week 3 with the same dose of vaccine candidate, then challenged successively at week 4 with CVB3 WT (G3) or CVB3/20 (G4) strains. Mice group 5 (G5) received no challenge. However, mice groups (G6 and G7) were prime-boosted with PBS at weeks 0 and 3, then challenged at week 4 with CVB3 WT (G6) or CVB3/20 (G7) strains. Mice group 8 (G8) represent the naive control non-inoculated mice. (**B**) Percentages of body weight changes and (**C**) survival among all mice groups (G1–G8) at different weeks post prime-immunization with vaccine candidate. (**D**) Viral loads in heart and (**E**) pancreas organ tissues of the different mice groups at the end of the study (Week 6). Viral titers of CVB3 WT and CVB3/20 strains were determined using HEK293T cell cultures in TCID_50_/mg. (**F**) Histology of hearts and pancreas tissues stained with HE and examined by light microscopy. Heart (F**1**) and pancreas (F**2**) tissues of mice groups challenged with CVB3 pathogenic strains. Heart (F**3**) and pancreas (F**4**) tissues of mice groups prime-boost immunized with rVP1 vaccine, then challenged with CVB3 pathogenic strains. Heart (F**5**) and pancreas (F**6**) tissues of naive control Balb/c mice group. Arrows indicate lymphocyte infiltrations in mice organ tissues. Magnification 40×. The data shown are the meaning of +/− SD from three replicated experiments (n = 3). * *p* < 0.05 compared vs. control, Student’s *t* test.

We evaluated the immunogenicity of the recombinant CVB3 VP1 vaccine candidate and we determined whether an immunization boost with the recombinant viral protein protected mice or not from virus challenges with WT and CVB3/20 pathogenic strains. The adopted immunization strategy is presented in Figure 3A. Balb/c mice groups 3 and 4 (G3 and G4) were immunized with 12 μg dose of vaccine candidate twice at day 0 and week 3. Mice groups were then successively challenged with pathogenic CVB3 WT and CVB3/20 strains at week 4-post prime-immunization. Groups 6 and 7 (G6 and G7) served as control non-immunized and non-boosted mice groups. However, both groups G6 and G7 were challenged at week 4 with pathogenic virus strains. Mice groups G1, G2, G5, and G8 were subjected to the same immunization strategy described in Section 3.2.

All mice groups were monitored daily during the study period for disease clinical signs, body weight changes (Figure 3B) and mortality (Figure 3C). Decreasing in body weight and mortality was used as indicator for viral severity infection. Following viral challenge, the average body weights of mice groups G6 and G7 declined substantially by weeks 5 and 6 post-immunization with PBS which correspond to weeks 1 and 2 post-challenge. However, mice of G6 and G7 rapidly lost considerably weight (more than 18%), due to the evolution of virus infection, making the animals sick and then eating less. Mice Group G5, receiving prime dose vaccination without any challenge showed moderate weight loss less than 5% in body weight. In contrast, we have not revealed a significant loss in mice body weight among groups G1–G4 prime-immunized or prime boost immunized with vaccine candidate. Control PBS group mice belonging to group G8 and prime-immunized group G5 mice non-challenged maintained their weights (Figure 3B).

Due to severe clinical signs and the rapid evolution of diseases within mice groups, we noted during the study the death of some mice. The percentage of mice survival was variable among the different groups, receiving or not lethal doses of CVB3 WT and CVB3/20 strains (Table S1, Supplementary Materials). Mice groups G1 and G2 prime-immunized and then challenged successively with the CVB3 WT lethal dose of 3.2 × 10^5^ TCID_50_ and with CVB3/20 lethal dose of 1.28 × 10^5^ TCID_50_ demonstrated a protective efficacy of 83%. However, mice groups G3 and G4 prime-boosted with vaccine dose and challenged with lethal doses of CVB3 WT and CVB3/20 strains showed in contrast a protective efficacy of 100%. The naive mice groups G6 and G7 challenged successively with lethal doses of CVB3 WT and CVB3/20 strains, and prime-boosted with PBS revealed, however, a protective efficacy of only 33%. These groups demonstrated the most elevated percentage of mortality in weeks 5 and 6 post-prime-immunization. However, naive PBS control mice groups G5 and G8 showed no significant mortality (Figure 3C).

In order to correlate the percentage of mice mortality with the pathogenic viral replication in mice, we evaluated the viral loads in heart and pancreas organ tissues, and we examined the histopathology of organ tissues of all mice groups at week 6 post-first immunization. All survival mice were euthanized at the end of the study. Hearts and pancreas organ portions were collected and CVB3 WT and CVB3/20 titers were calculated separately on HEK293T cell cultures, using the Reed and Muench assay. For histopathology examination, tissues were fixed in formalin for paraffin embedding and a serial 3–5 μm thick sections of different organ tissues were stained with HE and examined by light microscopy for evidence of pathologic damages. The viral titers were quantified by the tissue culture infectious doses in a given weight of organ tissues (TCID_50_/mg of tissues). Figure 3D,E present the viral yields successively in heart and pancreas tissues of all Balb/c mice groups G1 to G8. Interestingly, the viral loads in both heart and pancreas tissues among mice groups G3 and G4 prime-boosted with vaccine candidate then challenged with pathogenic CVB3 strains were significantly lower than among mice groups G1 and G2 prime-immunized with rVP1 vaccine candidate and challenged with CVB3 pathogenic strains. Indeed, viral titers in mice groups G3 and G4 showed a substantial reduction of 25% and 72% successively, compared to those in G1–G2 and G6–G7 mice groups. In addition, results of histology examination showed that CVB3 WT and CVB3/20 strains provoked widespread inflammatory lesions and significant extension of infiltration and necrosis areas (pancreatitis and myocarditis) in heart and pancreas muscles of successively mice Groups 6 and 7 non-immunized with rVP1 vaccine candidate (Figure 3(F1,F2)). However, hearts and pancreas tissues of mice Groups 3 and 4 prime-boost immunized with rVP1 vaccine and challenged successively with CVB3 WT and CVB3/20 strains (Figure 3(F3,F4)) appeared normal and indistinguishable from heart and pancreas from naive control mice Group 8 (Figure 3(F5,F6)). Taken together, results of tissues viral titration and histopathology examination demonstrated that the prime-boost immunization with the recombinant rVP1 vaccine candidate could enhance the protective immune responses and protect Balb/c mice from the lethal viral challenges. As expected, the viral titers of CVB3 WT and CVB3/20 strains in non-immunized and challenged mice groups G6–G7 were considerably high.

## 4. Discussion

For a long time, vaccines have been effectively used to reduce the incidence of viral infectious diseases. Recently, several technological advances have led to the conception of new generations of vaccines. Subunit based-vaccines, especially when combined with adjuvants, are qualified as safer, stable, and more immunogenic than conventional vaccines (live-attenuated and inactivated vaccines). Different expression systems have been used to generate subunit polypeptide vaccines, including baculovirus/insect cells, bacteria, plants, yeast, and mammalian cells without the need of propagating pathogenic viruses [23,24]. Some vaccines based on subunits against various infectious pathogens have already been licensed for human use. Expression of recombinant viral structural proteins can take place in various living cell expression systems after which the structural viral proteins can be produced and reconstructed. These recombinant viral proteins are highly immunogenic and can elicit both cellular and humoral immune responses [25].

CVB3, belonging to the genus of *Enterovirus* from the *Picornaviridae* family, is a ubiquitous infection agent that infects many organs in the human body systems [1,2,3]. The associations of CVB3 with heart diseases and type 1 diabetes (T1D) are evident. Several studies demonstrated that CVB3 infections are associated with acute viral pancreatitis, myocarditis, chronic viral cardiomyopathy (CMD), and type 1 diabetes (T1D). The mode of contamination of CVB3 strains is mainly the fecal oral route (digestive tract). Natural infection in humans and experimental infection in mice (intraperitoneal and oral routes) provoke the synthesis of both neutralizing antibodies and interferon gamma cytokine detected in the serum (systemic antibodies). Viral capsid protein 1 (VP1) is the most exposed and contains major neutralization epitopes. Several studies have shown the potential of this viral protein in both vaccine developing and viral infection diagnosis [26].

In the present study, we produced a recombinant viral protein rVP1 by the expression of the CVB3 wild type viral protein using the well-known Bac-to-Bac recombinant baculovirus system cultured in Sf9 insect cells. We have shown that the produced recombinant protein VP1 was highly purified by ultracentrifugation and ion exchange chromatography. When examined by several biochemical and biophysical methods (SDS–PAGE and WB), the generated rVP1 was similar in molecular weight to the native viral protein. Quality assessments confirmed that CVB3 rVP1 stocks generated are pure, with a sufficient yield, free of baculoviral gp64 glycoprotein and residual dsDNA. Additional assays, especially X-ray diffraction assays of the generated product, could provide further information, especially regarding the three-dimensional conformation of the expressed protein. Results of the quality control fitted well with acceptable limits, encouraging us to conduct mice immunization experiments using the generated rVP1 as a vaccine candidate.

The lethal challenge of Balb/c mice with two different pathogenic CVB3 WT and CVB3/20 strains were tested to evaluate the immune humoral and cellular responses. Using different strategies of immunization and lethal challenges, we demonstrated that immunization with the rVP1 induce effectively the synthesis of specific neutralizing Ab against both virus pathogenic strains and the production of IFN-γ cytokine. Interestingly, antibodies anti-VP1 produced in sera of different mice groups raised by rVP1 prime or prime-boost immunization exhibited a potent neutralizing activity against the two tested strains CVB3 WT and CVB3/20. Titers of specific neutralizing Ab anti-CVB3 WT and anti-CVB3/20 increased notably after the prime-boost immunization and remarkably after challenges with the two pathogenic strains. Our results of the specific humoral responses are very similar to several previous studies showing the same efficacy of viral protein VP1 as immunogens [4,16,27]. In this study, we evaluated the effect of the prime-boost regimen whereas in other studies, researchers used adjuvants. We demonstrated that specific neutralizing antibody titers and IFN-γ cytokine concentrations were increased without using any adjuvant, thus enhancing both humoral and cellular immune responses. However, the ability of the recombinant subunit vaccines to stimulate potent protective and long-term memory immune responses may be enhanced by immunostimulatory adjuvants that are often delivered along with vaccines. Integrating an adjuvant directly into the rVP1 vaccine could be a major advance for vaccine immunogenicity. Several studies demonstrated that the adjuvant delivery system enhances significantly vaccine immunogenicity by optimizing the efficient delivery of antigens, boosting immune cell activation, controlling antigen release rate, and prolonging the duration of immune responses (reviewed in [28]). In addition, we have evaluated the immunized and challenged mice groups body weight changes and the percentage of survival mice. Our results demonstrated that the weight loss among PBS control mice and challenged with CVB3 WT and CVB3/20 pathogenic strains was significant, making animals feel sick and then eating less. In contrast, we have not observed any body weight change among prime-boost immunized and challenged mice groups compared to the control mice group. Additionally, we examined the survival rates of the different mice groups studied. Our results highlighted that the prime-boost immunized and challenged mice groups demonstrated a protective efficacy of 100%. However, a notable death rates was observed among mice groups non-immunized and challenged with CVB3 WT and CVB3/20 pathogenic strains. Taken together, results highlighted that a prime-immunization and particularly a prime-boost immunization with the recombinant viral protein rVP1 could protect Balb/c mice against lethal challenges with pathogenic strains CVB3 WT and CVB3/20.

## 5. Conclusions

The present study presents results of the production of an effective and protective recombinant viral protein rVP1 vaccine candidate against CVB3 infections. We highlighted in this study the efficacy of the designed vaccine candidate to induce immune response and protect Balb/c mice against lethal challenges, making it as a potential vaccine candidate. However, further investigations regarding the expression of T cell CD4+ and CD8+ in lymph nodes and spleen could clarify more the mechanism of the immune response induced by this vaccine candidate.

## Data Availability

The data presented in this study is available on request from the corresponding author.

## Supplementary Materials

The following supporting information can be downloaded at: https://www.mdpi.com/article/10.3390/vaccines14030244/s1, Table S1: Percentages of mice survival among all groups (G1–G8) at different weeks post prime-immunization with the recombinant viral protein rVP1 vaccine candidate.
