# Supplementary material for: The Recombinant Viral Capsid Protein rVP1 Induces Protective Immunity Against Coxsackievirus B3 (CVB3) Lethal Challenges in Balb/c Mouse Model
Source: Vaccines (Basel). 2026 Mar 6;14(3):244. doi: 10.3390/vaccines14030244 (PMC13030759; doi:10.3390/vaccines14030244)
Supplement: Supplementary file 1 [file vaccines-14-00244-s001.zip › vaccines-4148861-supplementary.pdf]

## Supplementary Information

# The Recombinant Viral Capsid Protein rVP1 Induces Protective Immunity Against Coxsackievirus B3 (CVB3) Lethal Challenges in Balb/c Mouse Model

**Manel Ben M'hadheb, Ikbel Hadj Hassine, Mohammed A. Almalki, Mouna Hassine and Jawhar Gharbi**

**Table S1.** Percentages of mice survival among all groups (G1-G8) at different weeks post prime-immunization with the recombinant viral protein rVP1 vaccine candidate.

| Mice Group | Number of Survival Mice / Total Number (percentage) at Different Weeks Post prime-Immunization | | | | | |
|---|---|---|---|---|---|---|
| | Week 1 | Week 2 | Week 3 | Week 4 | Week 5 | Week 6 |
| G1 | 6/6 (100%) | 6/6 (100%) | 6/6 (100%) | 6/6 (100%) | 5/6 (83%) | 5/6 (83%) |
| G2 | 6/6 (100%) | 6/6 (100%) | 6/6 (100%) | 6/6 (100%) | 5/6 (83%) | 5/6 (83%) |
| G3 | 6/6 (100%) | 6/6 (100%) | 6/6 (100%) | 6/6 (100%) | 6/6 (100%) | 6/6 (100%) |
| G4 | 6/6 (100%) | 6/6 (100%) | 6/6 (100%) | 6/6 (100%) | 6/6 (100%) | 6/6 (100%) |
| G5 | 6/6 (100%) | 6/6 (100%) | 6/6 (100%) | 6/6 (100%) | 6/6 (100%) | 6/6 (100%) |
| G6 | 6/6 (100%) | 6/6 (100%) | 6/6 (100%) | 6/6 (100%) | 2/6 (33%) | 2/6 (33%) |
| G7 | 6/6 (100%) | 6/6 (100%) | 6/6 (100%) | 6/6 (100%) | 3/6 (50%) | 2/6 (33%) |
| G8 | 6/6 (100%) | 6/6 (100%) | 6/6 (100%) | 6/6 (100%) | 6/6 (100%) | 6/6 (100%) |
